# Community interventions for improving adult mental health: mapping local policy and practice in England

**DOI:** 10.1186/s12889-021-11741-5

**Published:** 2021-09-16

**Authors:** F. Duncan, C. Baskin, M. McGrath, J. F. Coker, C. Lee, J. Dykxhoorn, E. A. Adams, S. Gnani, L. Lafortune, J. B. Kirkbride, E. Kaner, O. Jones, G. Samuel, K. Walters, D. Osborn, E. J. Oliver

**Affiliations:** 1 Department of Sport and Exercise Sciences, Durham University, 42 Old Elvet, Durham, DH1 3HN UK; 2 Department of Primary Care and Public Health, Imperial College London, St Dunstan’s Road, London, W6 8RP UK; 3 Division of Psychiatry, University College London, 149 Tottenham Court Road, London, W1T 7BN UK; 4 Cambridge Public Health Interdisciplinary Research Centre, University of Cambridge, Robinson Way, Cambridge, CB2 0SR UK; 5 Department of Primary Care and Population Health, University College London, Rowland Hill Stress, London, NW3 2PF UK; 6 Population Health Sciences Institute, Newcastle University, Baddiley-Clark Building, Newcastle, NE2 4AX UK; 7 The McPin Foundation, 7-14 Great Dover Street, London, SE1 4YR UK; 8 Camden and Islington NHS Foundation Trust, London, NW10PE UK

**Keywords:** Public mental health, Wellbeing, Community interventions, Mapping exercise, Determinants of public mental health, Health policy

## Abstract

**Background:**

Public mental health (PMH) aims to improve wellbeing and prevent poor mental health at the population level. It is a global challenge and a UK priority area for action. Communities play an important role in the provision of PMH interventions. However, the evidence base concerning community-based PMH interventions is limited, meaning it is challenging to compare service provision to need. Without this, the efficient and equitable provision of services is hindered. Here, we sought to map the current range of community-based interventions for improving mental health and wellbeing currently provided in England to inform priority areas for policy and service intervention.

**Method:**

We adopted an established mapping exercise methodology, comparing service provision with demographic and deprivation statistics. Five local authority areas of England were selected based on differing demographics, mental health needs and wider challenging circumstances (i.e. high deprivation). Community-based interventions were identified through: 1) desk-based research 2) established professional networks 3) chain-referral sampling of individuals involved in local mental health promotion and prevention and 4) peer researchers’ insight. We included all community-based, non-clinical interventions aimed at adult residents operating between July 2019 and May 2020.

**Results:**

407 interventions were identified across the five areas addressing 16 risk/protective factors for PMH. Interventions for social isolation and loneliness were most prevalent, most commonly through social activities and/or befriending services. The most common subpopulations targeted were older adults and people from minority ethnic backgrounds. Interventions focusing on broader structural and environmental determinants were uncommon. There was some evidence of service provision being tailored to local need, though this was inconsistent, meaning some at-risk groups such as men or LGBTQ+ people from minority ethnic backgrounds were missed. Interventions were not consistently evaluated.

**Conclusions:**

There was evidence of partial responsiveness to national and local prioritising. Provision was geared mainly towards addressing social and individual determinants of PMH, suggesting more integration is needed to engage wider service providers and policy-makers in PMH strategy and delivery at the community level. The lack of comprehensive evaluation of services to improve PMH needs to be urgently addressed to determine the extent of their effectiveness in communities they serve.

**Supplementary Information:**

The online version contains supplementary material available at 10.1186/s12889-021-11741-5.

## Introduction

Globally, the magnitude of the mental ill health burden is not matched by the size and effectiveness of the response it demands. Each year in England, almost a quarter of all adults experience at least one mental health condition [1]. Poor mental health has a profoundly negative impact on life expectancy [2] and quality of life [3], and increases the risk of physical illness [4–6]. It has also wider societal impacts such as work absence [7], unemployment [8], and homelessness [1]. Yet, there has been chronic underinvestment in mental health care across the National Health Service (NHS) [9], as well as in early identification and prevention of mental illness [10–12] and in promoting positive mental health and wellbeing [12]. The challenge across these areas has been exacerbated by austerity measures imposed in the UK since 2010 [10, 11]. There is some evidence that the COVID-19 pandemic has had negative consequences for mental health and well-being in the short-term [13–15] and are likely to be exacerbated by secondary economic and social stressors. It is clear that more effective intervention is needed to support both individual and population level mental health.

Public mental health promotion aims to improve wellbeing and prevent mental ill health at the population level. Currently, in the UK, the approach is through coordinated work with a variety of public, third sector organisations, local communities and individuals [1]. There are a wide range of risk factors for poor mental health that public mental health approaches seek to address, many of which lie outside the remit of health services. These include economic disadvantage [1, 16], debt and financial difficulties [17–20], unemployment [8], social isolation and loneliness [8], intimate partner violence [21], sedentary lifestyles [22–24], food insecurity [25, 26] homelessness [27] and discrimination [8]. Many of these factors have been worsened by the COVID-19 pandemic [28–31]. Public mental health approaches also seek to support a range of protective factors for good mental health such as community safety and cohesion and the promotion of physical activity and other health behaviours [12, 32, 33].

Community-based interventions that seek to support mental health and wellbeing, although not consistently defined [34–38], include a broad range of non-clinical programmes generally targeting both individuals and the communities in which they live, from individual support and practical assistance, through to mobilising community connections and resources. There is emerging evidence that community-based interventions that tackle the social determinants of mental health and wellbeing have the potential to improve resilience, mental health outcomes, and the psychosocial circumstances of individuals and the wider community [1, 12].

However, the current evidence base for effectiveness and cost-effectiveness of community-based interventions is limited [39, 40]. For example, the wide range of interventions and outcome measures for older adults, confounds a clear understanding of what works [41]. While some interventions have shown promise for adults of working age, such as workplace-based stress management [42], co-located welfare advice [43], group job-skills training, and social prescribing [40] there is generally little understanding of the underlying mechanism of effect, limiting efforts to replicate and scale-up successful practices. Interventions are often complex combinations of components, diverse in both form and function [41]. At present, our knowledge of the full range of interventions currently being delivered within local communities is limited, with systematic evidence reviews generally only reporting comprehensively evaluated interventions. Indeed, a recent scoping review [44] identified, and excluded due to inadequate evaluation, over 50 community interventions that focused on mental health in ethnic minority adults in the grey literature, highlighting the breadth of available services that are not yet being rigorously evaluated, with opportunities for learning missed.

Given the limited evidence base, it is therefore challenging to compare service provision to need, to identify where public mental health interventions have been comprehensively deployed and where there might be opportunities for enhancement. Without this, the efficient and equitable provision of services, and shared learning around what is effective (or ineffective) and for whom, is hindered. Thus, a better understanding of the types of community-based interventions currently delivered for public mental health is important for policy makers and commissioners. This would highlight gaps in the current evidence base and promising areas for future research and evaluation. It is important to consider key components, context and sustainability, and how to best develop them, given varied needs and assets across communities. This is especially important since the COVID-19 pandemic as it is anticipated that there will be a greater need for such services in the coming months and years.

In light of this evidence gap, this study aimed to 1) identify the range of community-based interventions for improving mental health and wellbeing currently provided in localities with different sociodemographic characteristics and needs, 2) describe the approach, target population, content and outcomes of each intervention and in doing so, 3) identify priority areas for policy and service intervention by critiquing findings against evidence of need in the localities selected, alongside conceptual models of the determinants of public mental health.

## Methods

We adopted a mapping exercise methodology, employed in other areas of population health, including childhood obesity, drug treatment services, and mental health [45–47]. A detailed protocol for this work has been published [48]. Mapping involves a structured process to systematically identify and collect information about interventions in a given area. It allows insight into how and whether resources are being used efficiently by mapping the availability of interventions to population need, highlighting who is accessing support, and importantly, who does not have access to valuable community assets. The information collected can inform the planning and commissioning of services, identify gaps in delivery and provisions, interventions that warrant future research and evaluation, and compare provision across localities [46].

### Inclusion and exclusion criteria

We defined, with stakeholder and peer researcher input, community-based interventions as any non-clinical programme, service or policy that explicitly sought to promote the mental health and wellbeing of residents. We included all community-based interventions aimed at adult residents (16 years and older) that were operating within the boundaries of five study localities during our period of data collection (July 2019 – May 2020). We excluded interventions that provided clinical care for individuals (e.g. pharmaceutical interventions or psychotherapy), as well as those aimed solely at children under 16 years.

### Phase 1: selection of case study localities

We purposively selected five study localities, covering approximately 1,825,000 people, using the following criterion: social deprivation, geographical location, rurality, age, and ethnic composition (Table 1). Criteria were not differentially weighted; candidate sites were discussed across the research team including with wider stakeholders. In selecting our final sites, we sought variation and diversity between the localities to ensure that a range of sub-populations, and local authority systems, were represented. This was helpful when considering whether and how approaches to the delivery of public mental health interventions differed depending on the specific sub-population, mental health needs, or the overall demographics of an area. Two study localities – the London Boroughs of Camden and Islington, and Cambridgeshire and Peterborough comprise two and six local authorities respectively but were treated as single study locality as their local government services, including their public health teams, work across local authorities. Characteristics of the case study localities are summarised in terms of population demographics, deprivation-related data (Table 1), and mental ill health prevalence (Table 2).

**Table 1:** Characteristics of selected study localities

	Region of England	Population^a^	Median age^a^	Over 65 years old^a^	Minority Ethnic^b^	Non-UK Born^c^	Living in rural areas^d^	IMD 2019^e^ – Extent^g^: Proportion of LSOAs^f^ in most deprived 30% nationally	IMD – Rank of Extent^h^: Rank of proportion of LSOAs in most deprived 30% nationally (1 = most deprived; 317 = least deprived)
Blackburn with Darwen	North West	148,942	36.3	14.5%	30.8%	15.6%	4.7%	0.55	7
Cambridgeshire	East	651,482	41.0	19.2%	7.4%	15.7%	64.6%		
- Cambridge								0.05	202
- East Cambridgeshire								0.00	295
- Fenland								0.21	96
- Huntingdonshire								0.02	250
- South Cambridgeshire								0.00	288
Camden	London	262,226	33.9	12.0%	33.7%	46.9%	0.0%	0.14	139
Hammersmith and Fulham	London	185,426	34.8	11.0%	31.9%	43.3%	0.0%	0.16	124
Islington	London	239,142	32.1	8.9%	32.0%	33.1%	0.0%	0.27	74
Peterborough	East	201,041	36.5	14.9%	16.7%	23.5%	12.3%	0.37	40
Redcar & Cleveland	North East	136,718	45.0	22.6%	1.4%	3.0%	32.5%	0.36	43
England	**…**	**55,977,178**	**39.9**	**18.4%**	**14.0%**	**15.5%**	**23.6%**	**N/A**	**N/A**

^a^Office of National Statistics UK mid-year estimates 2018/2019 [49]

^b^Office of National Statistics Census 2011 [50].

^c^Office of National Statistics Population by country of birth and nationality July 2018 to June 2019 [51]

^d^Office of National Statistics Census 2011 RUC11_LAD11_Env2 [52]

^e^Deprivation data from English Indices of Deprivation 2019 [53]. The IoD2019 is comprised of seven distinct domains of deprivation which, when combined and appropriately weighted, form the Index of Multiple Deprivation 2019. Income (22.5%), Employment (22.5%), Health Deprivation and Disability (13.5%), Education, skills and training (13.5%), Crime (9.3%), Barriers to Housing and Services (9.3%), Living Environment (9.3%)

^f^LSOAs (Lower-Layer Super Output Area) are standard areas created for the purpose of statistical analysis. There are 32,844 LSOAs in England, with an average population of 1500. Each LSOA in England is ranked from most deprived (rank of 1) to least deprived (rank of 32,844) [53]

^g^Extent – The “extent” measure is a summary of the proportion of the local population that live in areas classified as among the most deprived in the country. The “extent” measure uses a weighted measure of the population in the most deprived 30% of all areas. The population living in the most deprived 10% of LSOAs in England received a “weight” of 1.0. The population living in the most deprived 11 to 30% of LSOAs receive a sliding weight, ranging from 0.95 for those in the most deprived eleventh percentile, to 0.05 for those in the most deprived thirtieth percentile. This provides an indication of the extent of deprivation in each local authority area [54].

^h^Rank of Extent – Once the “extent” measure has been calculated as described, the local authority areas are ranked from most deprived (a rank of 1) to least deprived (a rank of 317) on this measure producing the “rank of extent” summary measure [54]

**Table 2:** Mental health characteristics of the selected study localities

	Deaths by suicide (2017–2019)^a^	Age-standardised suicide rates per 100,000 population (2017–2019)^a^	Years of life lost to suicide age-standardised rate 15–74 years per 10,000 population (3 year average) (Persons) 2017–2019^b^	Estimated prevalence of common mental disorders: % of population aged 16 and over (2017)^c^	Estimated prevalence of common mental disorders: % of population aged 65 and over (2017)^c^
Blackburn with Darwen	33	8.3	32.3	19.9^d^	12.0
Cambridgeshire	178	10.4	33.0	14.2^e^	8.9
Camden	69	11.3	28.5	19.4^d^	11.8^d^
Hammersmith and Fulham	50	11.0	30.0	20.4^d^	12.5^d^
Islington	54	10.4	25.3	22.7^d^	13.8^d^
Peterborough	63	12.4	39.6	18.8^d^	11.3
Redcar & Cleveland	47	13.5	49.3^d^	18.1^d^	11.4
England	**14,788**	**10.1**	**33.0**	**16.9**	**10.2**

^a^ONS Suicides in England and Wales by Local authority [55].

^b^Public Health England. Public Health Profiles. Mental Health Dementia and Neurology. Suicide Prevention [56].

^c^Public Health England. Public Health Profiles. Mental Health Dementia and Neurology. Mental Health and Wellbeing JSNA [57]

^d^Significantly higher than for England

^e^Significantly lower than for England

Sites were:

- **Blackburn with Darwen Council** is a unitary authority located in Lancashire in North West England.. It consists of the towns of Blackburn and Darwen as well as some villages. **2. Cambridgeshire and Peterborough** is a combined authority located in the East of England. **3. London Boroughs of Camden and Islington** comprise two distinct local authorities that share a public health team and are located in central London.**4. London Borough of Hammersmith and Fulham** is a unitary authority located in the West of London. **5. Redcar and Cleveland Borough Council** is a unitary authority located within Tees Valley in the North East of England.. It is a coastal borough and consists of a collection of larger and smaller towns.

### Phase 2: data collection and analysis

A data collection template was developed based on the TIDieR (Template for Intervention Description and Replication) Checklist [58], with some adaptation to increase its applicability to public health interventions (Additional file). The template was piloted in two study sites (Redcar and Cleveland, and Camden and Islington) before being refined following discussion within the research team.

The data collection process was standardised to ensure the same degree of engagement with sites irrespective of the quality of pre-existing relationships between local stakeholders and research team members. Each search began with desk-based research. The websites of local authorities, NHS Clinical Commissioning Groups, and relevant third sector organisations were searched for policy documents, annual reports, strategies, and guides signposting local residents to interventions in their area. A standardised list of key informants in each study site were then contacted to provide further information about each intervention, identify any missing interventions, and connect the research team to other relevant organisations in their area. Key informants included: public health practitioners, commissioners, programme managers, local link workers, community navigators, and representatives from voluntary sector organisations. Data collection with key informants involved multiple methods, including telephone and email, face-to-face meetings, an online questionnaire, attending local mental health committees and analysis of any documentation they provided. Importantly, peer researchers either with knowledge of case study sites and/or skills to undertake community research, contributed to data collection and intervention identification. While it cannot be known if all interventions were captured, the researchers continued to search until they had an exhaustive list of programmes identifiable through desk-based research and community contacts.

### Phase 3: developing the coding framework

To allow comparability across sites and between interventions, a coding framework was developed by the primary data collectors (FD, MM, CB, JC) iteratively to categorise intervention type, risk or protective factors, and target population. In order to ensure consistency across regions, a selection of the same interventions was individually coded by the primary researcher from each area. Discrepancies in coding were then discussed across the research team until consensus was reached. We present summary statistics and a narrative synthesis of results.

Ethical approval for the research was granted by the Department of Sport and Exercise Science Research Ethics Committee at Durham University (Reference: SPORT-2019-06-28 T15:10:42-lxkc61). This study is part of a larger National Institute of Health Research (NIHR) funded programme through the School for Public Health Research (SPHR) and a study protocol has been published [48].

## Results

A total of 407 interventions meeting our inclusion criteria were identified across the five study sites: Camden and Islington (*n* = 65; 15.9%), Hammersmith and Fulham (*n* = 75; 18.4%), Cambridgeshire and Peterborough (*n* = 106; 26%), Redcar and Cleveland (*n* = 62; 15.2%) and Blackburn with Darwen (*n* = 99; 24.3%). Data collection for four of the five localities was completed before the start of the COVID-19 pandemic restrictions in March 2020. Data collection for Cambridgeshire and Peterborough continued until May 2020, however focused entirely on interventions offered prior to the start of the national restrictions.

Table 3 provides a breakdown of the types of intervention and risk/protective factors by area. Descriptions of the risk/protective factors that the interventions aimed to address and intervention types are provided in Tables 4 and 5 respectively.

**Table 3:** Number of interventions categorized by type and risk/protective factor per selected area

	Camden and Islington	Hammersmith and Fulham	Cambridgeshire and Peterborough	Redcar and Cleveland	Blackburn with Darwen	Total
**Type of Intervention**	*N =* 65 (15.9%)	*N =* 75 (18.4%)	*N =* 106 (26.0%)	*N =* 62 (15.2%)	*N =* 99 (24.3%)	***N*** **= 407**
Signposting, information referral, advice services	**36 (55.4%)**	**28 (37.3)**	**23 (21.7%)**	**18 (29%)**	**36 (36.4%)**	**141 (34.6%)**
Advocacy and legal support	**20 (30.8%)**	**8 (10.7%)**	**5 (4.7%)**	**1 (1.6%)**	**3 (3%)**	**37 (9.1%)**
Education, training and workshops to expand skillsets	**23 (35.4%)**	**19 (25.3%)**	**19 (17.9%)**	**18 (29%)**	**18 (18.2%)**	**97 (23.8%)**
Education, training and workshops for mental health awareness, prevention and recovery	**10 (15.4%)**	**17 (22.7%)**	**7 (6.6%)**	**12 (19.4%)**	**27 (27.3%)**	**73 (17.9%)**
Promoting physical activity	**13 (20%)**	**8 (10.7%)**	**3 (2.8%)**	**5 (8.1%)**	**14 (14.1%)**	**43 (10.6%)**
Peer support, mentoring	**19 (29.2%)**	**20 (26.7%)**	**28 (26.4%)**	**18 (29%)**	**24 (24.2%)**	**109 (26.8%)**
Social activities and befriending	**32 (49.2%)**	**33 (44%)**	**75 (70.8%)**	**21 (33.9%)**	**21 (21.2%)**	**182 (44.7%)**
Practical help	**8 (12.3%)**	**10 (13.3%)**	**11 (10.4%)**	**2 (3.2%)**	**6 (6.1%)**	**37 (9.1%)**
Food security interventions	**4 (6.2%)**	**5 (6.7%)**	**0 (0%)**	**1 (1.6%)**	**1 (1.01%)**	**11 (2.7%)**
Policies, strategies, funding and networks	**9 (13.8%)**	**9 (12%)**	**7 (6.6%)**	**8 (12.9%)**	**19 (19.2%)**	**52 (12.8%)**
Animal and green space interventions.	**1 (1.5%)**	**1 (1.3%)**	**4 (3.8%)**	**1 (1.6%)**	**1 (1.01%)**	**8 (2.0%)**
Prevention of further decline in mental illness	**10 (15.4%)**	**6 (8%)**	**7 (6.6%)**	**3 (4.8%)**	**4 (4.04%)**	**30 (7.4%)**
**Risk or Protective Factor**						
Financial stress	**23 (35.4%)**	**19 (25.3%)**	**4 (3.8%)**	**5 (8.1%)**	**9 (9.1%)**	**60 (14.7%)**
Job insecurity and unemployment	**9 (13.8%)**	**7 (9.3%)**	**5 (4.7%)**	**9 (14.5%)**	**7 (7.1%)**	**37 (9.1%)**
Mental health stigma, knowledge and awareness	**12 (18.5%)**	**13 (17.3%)**	**7 (6.6%)**	**7 (11.3%)**	**14 (14.1%)**	**53 (13%)**
Stigma, discrimination, marginalisation – Ethnicity and migration status	**18 (27.7%)**	**22 (29.3%)**	**4 (3.8%)**	**0 (0%)**	**7 (7.1%)**	**51 (12.5%)**
Stigma, discrimination, marginalisation – LGBTQ+	**5 (7.7%)**	**3 (4%)**	**1 (0%)**	**0 (0%)**	**6 (6.1%)**	**14 (3.4%)**
Social isolation and loneliness	**27 (41.5%)**	**28 (37.3%)**	**68 (64.2%)**	**20 (32.3%)**	**13 (13.1%)**	**156 (38.3%)**
Caring responsibilities	**5 (7.7%)**	**3 (4%)**	**4 (3.8%)**	**4 (6.5%)**	**4 (4.04%)**	**20 (4.9%)**
Community safety and cohesion	**9 (13.8%)**	**10 (13.3%)**	**12 (11.3%)**	**5 (8.1%)**	**7 (7.1%)**	**43 (10.6%)**
Food insecurity	**3 (4.6%)**	**4 (5.3%)**	**0 (0%)**	**2 (3.2%)**	**1 (1.01%)**	**10 (2.5%)**
Gender-based violence	**8 (12.3%)**	**12 (16%)**	**0 (0%)**	**1 (1.6%)**	**3 (3.03%)**	**24 (5.9%)**
Access to health and social services	**7 (10.8%)**	**19 (25.3%)**	**2 (1.9%)**	**1 (1.6%)**	**4 (4.04%)**	**33 (8.1%)**
Bereavement	**3 (4.6%)**	**1 (1.3%)**	**5 (4.7%)**	**2 (3.2%)**	**6 (6.1%)**	**17 (4.2%)**
Family relationships and Parenting	**4 (6.2%)**	**7 (9.3%)**	**5 (4.7%)**	**5 (8.1%)**	**5 (5.1%)**	**26 (6.4%)**
Physical activity and other health behaviours	**8 (12.3%)**	**7 (9.3%)**	**1 (0.9%)**	**9 (14.5%)**	**18 (18.2%)**	**43 (10.6%)**
Mood, confidence and self-esteem	**7 (10.8%)**	**0 (0%)**	**14 (13.2%)**	**13 (21%)**	**15 (15.2%)**	**49 (12%)**
All risk factors/Risk factor not specified/Other	**6 (9.2%)**	**7 (9.3%)**	**12 (11.3%)**	**11 (17.7%)**	**14 (14.1%)**	**50 (12.3%)**

**Table 4:** Descriptions of PMH risk factors/protective factors that identified community-centred interventions aimed to address

Risk factor / protective factor	Description
Financial stress	Psychosocial problems relating to debt, finances, welfare benefits payments, insecure housing, and employment.
Job insecurity and unemployment	Programmes for unemployed people or people in insecure job positions to progress toward stable employment.
Mental health stigma, knowledge and awareness	Stigma and discrimination experienced by people with mental health problems, limited awareness, skills and knowledge about mental health.
Stigma, Discrimination and Marginalisation - Ethnicity and migration status	Problems specific to minority ethnic groups (e.g. stigma, discrimination, migration-related difficulties, language barriers, refugee and immigration status)
Stigma, Discrimination and Marginalisation – LGBTQ+	Problems specific to LGBTQ+ groups (e.g. stigma, discrimination, limited awareness of trans and non-binary)
Social isolation and loneliness	Limited social interactions, feeling alone/unsupported or socially disconnected.
Caring responsibilities	Difficulties experienced by people due to their caring role (e.g. burnout, reduced employment opportunities, financial stress)
Community safety and cohesion	Experiences and perceptions of crime, violence and safety in their neighbourhood (e.g. gangs, knife crime, anti-social behaviour, hate crimes), efforts to build social cohesion and community engagement.
Food insecurity	Unreliable or insufficient availability of food
Gender-based violence	Actual or threatened physical, sexual, emotional or financial violence committed against women (e.g. intimate partner violence, sexual harassment and intimidation, female genital mutilation, forced marriage)
Access to health and social services	Difficulties accessing and navigating the health and social care system, including issues around the availability, cultural acceptability and affordability of services.
Bereavement	Grief, bereavement and traumatic loss following the death of friends or family.
Family relationships and parenting	Stable and supportive family relationships, divorce and separation, household conflict, problems experienced in pregnancy or during the transition to parenthood, and difficulties providing for your children.
Physical activity and other health behaviours	The promotion of health behaviours that encourage positive mental health, including increased physical activity and reduced alcohol and drug use.
Mood, confidence and self-esteem	Feeling low in mood, anxious, stressed, depressed, worried, angry and lacking in confidence or self-esteem.
All risk factors/Risk factor not specified/Other	Interventions which does not aim to target any specific risk factor, where it aims to target all or any risk factor or where the risk factor does not belong in any of the above categories.

**Table 5:** Descriptions of the types of PMH community-centred interventions identified in mapping exercise

Type of intervention	Description
Signposting, information referral, advice services	Linking residents to non-clinical sources of support, advice and information. This includes what is often described as “social prescribing”.
Advocacy and legal support	Active support, advocacy and case management provided to residents.
Education, training and workshops to expand skillsets	Training courses and workshops to develop skills and increase confidence (e.g. job interview training, IT skills development, financial literacy workshops, art courses)
Education, training and workshops for mental health awareness, prevention and recovery	Training courses, workshops and self-help material that aim to prevent suicide, help residents manage stress and anxiety (e.g. stress management courses, relaxation workshops and wellbeing apps, online self-help guides, helplines), maintain good mental health (e.g. ‘The Recovery College’), increase knowledge around mental health (e.g. mental health first aid) and reduce stigma associated with mental illness.
Promoting physical activity	Programmes designed to increase physical activity of residents with the aim of promoting positive mental health (e.g. walking groups, dance classes, yoga)
Peer support and mentoring	Help, guidance and reciprocal support offered by peers, volunteers, or other members of the community.
Social activities and befriending	Events and groups that aim to connect residents with others in their community to reduce social isolation and build confidence (e.g. community events, sports groups, art classes, befriending services)
Practical help and assistance	Practical help designed to improve the quality of life of residents and support independent living. This includes ensuring access to affordable household heating, improving household security and fire safety, conducting falls assessments, assisting with transport, cleaning, picking up prescriptions, groceries and baby supplies, and providing respite for carers and baby banks that provide baby necessities to parents.
Food security interventions	Programmes that promote wellbeing by providing meals, groceries, practical skills and support to those experiencing food insecurity.
Policies, strategies, funding and networks	Initiatives that aim to encourage collaboration and joint working, promote the mental health and wellbeing “agenda”, provide small grants, build organisational and community capacity (including supporting community groups and training volunteers), reduce fragmented referral care pathways or improve the delivery and implementation programmes.
Animal and green space interventions	Programmes designed to promote positive mental health through increased access to calming spaces for residents (e.g. greenspace initiatives, quiet rooms, animal farms).
Prevention of further decline in mental illness	Interventions which aim to prevent decline of those who already have poor mental health

### Intervention type

Twelve types of intervention were identified (Table 3). Across all localities the most common type of intervention was social activities and/or befriending (*n* = 182; 44.7%); followed by signposting, information referral and advice services (this includes what is often described as “social prescribing”) (*n* = 141; 34.6%); peer support and mentoring (*n* = 109; 26.8%); and education, training and workshops to expand skillsets (*n* = 97; 23.8%). The least common types of intervention identified were food security interventions (*n* = 11; 2.7%) and animal and green space interventions (*n* = 8; 2%). The majority of interventions across all five localities consisted of a combination of key interventions (29.7% consisted two, 16.7% three, 10.3% four or more) and therefore were coded as more than one type.

### Risk or protective factor

The identified interventions across all five localities aimed to address 16 risk or protective factors (Table 3). We have not distinguished between risk factors and protective factors in the analysis as some of our codes could be considered to be both a risk factor and a protective factor. For example, family relationships can be destructive or supportive and can therefore either protect a person from poor mental health or contribute to poor mental health. The most common risk/protective factor was social isolation and loneliness (*n* = 156, 38.3%); followed by financial stress (*n* = 60, 14.7%); mental health stigma, knowledge and awareness (*n* = 53, 13%); and stigma, discrimination and marginalisation –ethnicity and migration status (*n* = 51; 12.5%). The least common risk/protective factors identified were caring responsibilities (*n* = 20; 4.9%); bereavement (*n* = 17; 4.2%); stigma, marginalisation, discrimination – LGBTQ+ (*n* = 14; 3.4%) and food insecurity (*n* = 10; 2.5%).

The majority of interventions aimed to address more than one risk factor or protective factor (31.2% aimed to address two risk factors, 10.8% three, 8.6% four or more) and therefore were coded as more than one type.

### Target population

The largest number of interventions were designed to support all residents within their respective localities (*n* = 167). The most common types of targeted populations were: older adults (*n* = 78), people from minority ethnic backgrounds (*n* = 52), women (*n* = 33), men (*n* = 27), carers (*n* = 21), and LGBTQ+ people (*n* = 13).

Very few interventions specifically supported marginalised groups at the intersection of ethnicity, gender and/or sexual orientation. The most commonly supported subgroup were minority ethnic women (*n =* 13). Few interventions supported minority ethnic men (*n =* 1) and LGBTQ+ people of minority ethnic background (*n =* 2) across all five localities.

### Priority areas for policy and service intervention – comparison of availability of PMH interventions with local need

All five localities contained LSOAs which were ranked in the 30% most deprived in England. As these rankings are constructed according to a range of indices of deprivation including income; employment; education, skills and training; health and disability; crime; barriers to housing and services; living environment deprivation, this suggests that residents in these localities may be exposed to a variety of drivers of poor mental health. It was clear from the mapping exercise that across all five localities some of these drivers featured in the service response e.g. through delivery of financial stress, job insecurity and unemployment and education, skills and training interventions services. However, services addressing other aspects of deprivation strongly associated with PMH, especially the delivery of housing services, did not feature (strongly) in the mapping.

There were a few notable differences in provision between localities. A larger proportion of residents in Cambridgeshire are over the age of 65 compared to the national average. Cambridgeshire also has a large rural population. This corresponds to a seemingly strong focus on interventions that aim to address social isolation and loneliness with social activities and/or befriending. The mapping of Cambridgeshire and Peterborough indicated a paucity of interventions for financial stress, gender-based violence, physical activity and other health behaviours, mental health stigma, knowledge and awareness and LGBTQ discrimination in this area. Lastly, Peterborough, Fenland district in Cambridgeshire and Redcar and Cleveland have higher suicide rates than the national average. We found a large number of interventions that aimed to address mood, self-confidence and self-esteem in Cambridgeshire and Peterborough and interventions that supported men as part of a suicide prevention strategy in Redcar and Cleveland.

### Delivery, funding and evaluation

The interventions were delivered, either independently or in partnership, by local authorities, NHS Clinical commissioning groups, third sector organisations and community groups. Stakeholders indicated that many of the interventions had not been comprehensively evaluated, potentially due to the short length of time they had been running or lack of funding for this purpose. This means we were not able to collect data on intervention outcomes, effectiveness or who and how many people the interventions reached. We were also unable to consistently obtain information on cost, funding history and financial sustainability of interventions. Several providers indicated uncertainty regarding whether their interventions would be funded in the long term or even beyond the current financial year.

## Discussion

This mapping exercise confirmed that a wide range of community-based interventions targeting improved public mental health are currently being delivered across England, showing some evidence of being tailored to key drivers of PMH locally. Assessing this was challenging, and determining effectiveness was not possible, given we were unable to consistently collect information of an evaluative nature, including: measurement of outcomes; costs; funding; participant numbers; and how long interventions had been running. Across localities there was strongest provision for social isolation and loneliness, most commonly through social activities and/or befriending services, yet broader interventions focusing on wider structural determinants are uncommon. There appeared to be some tailoring of services according to the relative size of population sub-groups.

### Determinants of public mental health: do the services available respond to drivers of PMH?

When compared with our conceptual work exploring the determinants of both poor and positive public mental health across the life course [59] this mapping exercise suggests that provision of public mental health interventions is currently limited to addressing a small number of these determinants. Across the five localities, the provision of services focused on addressing only a small number of the risk and protective factors, principally, the individual and their social environment. We found very few interventions focused on other broader determinants of mental health and wellbeing, including issues surrounding housing, retirement, and family circumstances. We did not identify interventions aimed at structural and environmental determinants, such as air and water quality, population density, walkability of local environment and urban decay, economic recession, climate change, natural disasters, media and advertising, the welfare system and political structures.

One explanation for the disparity between PMH determinants and service provision is that some risk or protective factors may be considered by local authorities and third sector organisations to be more important than others, therefore there may not be a requirement to have specific interventions or strategies targeting every determinant. For example, if communities can simultaneously tackle social isolation, loneliness and financial stress, then this may create a solid foundation for individuals to build resilience to other problems. Evidence suggests that social support may moderate environmental vulnerabilities and confer resilience to stress [60, 61]. However, the evidence base is insufficient to support a hierarchical approach to tackling risk and protective factors. Furthermore, there is likely to be coalescence, for instance, between loneliness and other aspects of deprivation.

The high number of services captured offering social activities and/or befriending may reflect the recent growth and prominence of social isolation and loneliness in UK government campaigns and high profile third sector organisations making it a priority for decision-makers (e.g., Let’s Talk Loneliness Campaign; Campaign to End Loneliness; Co-op Loneliness Campaign; Country Living Loneliness Campaign; Age UK campaigns). For example, the England pilot region for the Campaign to End Loneliness was Cambridgeshire and Peterborough where the highest number of social isolation and loneliness interventions were identified in the mapping exercise. This suggests that the risk factors/protective factors and subpopulations ‘targeted’ by these interventions may reflect political will and council priorities [62]. It is also plausible that social activities and befriending services were so common across all five localities because of their ease of provision and relatively low cost of delivery compared to more complex and expensive interventions that require a skilled workforce such as advice services, advocacy and legal support or education and training interventions.

We consider it likely that this study did not map interventions aimed at structural and environmental determinants because they did not explicitly state that they targeted mental health outcomes. This may indicate that providers of these broader services do not view themselves as playing a PMH role and/or are not involved with strategic discussions around PMH. It is also possible that we did not find evidence of these determinants of PMH being addressed at the community level because local authorities and third sector organisations do not currently have the resources, authority, influence, or power that is required to deliver such services. If this is the case, then consideration should be given to expanding the scope and abilities of community-led responses as evidence suggests that community approaches play an important role in increasing people’s self-efficacy, confidence, helping them to develop a sense of control over their own lives, reducing health inequalities, improving health outcomes and increasing resident’s sense of wellbeing [63, 64]. Therefore, there may be benefits to developing community-based interventions to tackle as many determinants of PMH as possible. If drivers associated with structural and environmental determinants are deemed important to address at the community level, then greater integration of policy and practice may be required, and reflected in engagement of a wider range of stakeholders from strategy to delivery and receipt of support.

### Universal and targeted interventions: gaps in provision of targeted interventions

The mapping exercise identified universal interventions that are open to all residents, as well as interventions targeted at specific groups in the community. For optimal improvements in PMH it is important to find the right balance between these two approaches [65]. Universal interventions have the advantages of being less stigmatising, less vulnerable to funding cuts and potentially easier to deliver than targeted interventions. However, they lack focus on the individualised needs of high-risk groups. Targeted interventions on the other hand can be tailored to the precise needs of vulnerable groups, which means fewer resources deployed on people who are unlikely to develop poor mental health [65] and therefore avoid further enabling advantage and increasing inequalities.

We found that the highest proportion of targeted interventions were for older adults. These interventions predominantly aimed to tackle social isolation and loneliness, especially in the more rural areas of Redcar and Cleveland and Cambridgeshire. We know that older adults and people living in rural areas have an increased risk of experiencing social isolation and loneliness [66]. However, we also know that older adults have needs other than social isolation and loneliness such as financial stress [67], physical activity for wellbeing [68] and bereavement [69]. Whilst targeted interventions for these challenges were identified in one area, in others they were not.

The second highest proportion of targeted interventions identified in the mapping exercise were aimed at people from a minority ethnic background. We know that those from minority ethnic groups are disproportionately affected by poor mental wellbeing, likely as a result of long-standing discrimination and disadvantage [44], therefore the existence of these interventions is encouraging as it indicates that attempts are being made to address the needs of these sub-populations. However, further targeting based on additional stressors was rare. There were very limited interventions found that specifically focused on men from minority ethnic backgrounds and these were only found in the study sites based in London. This is concerning given the evidence that men of minority ethnic background are less likely to seek help for common mental health problems [70, 71] and the evidence that culturally sensitive interventions are more likely to lead to positive outcomes [72].

With respect to other potentially underserved populations, there are a few notable gaps in provision. The mapping exercise revealed that there are a very limited number of interventions specifically aimed at LGBTQ+ people of minority ethnic background. This is concerning as people who belong to multiple minority groups are particularly vulnerable to developing poor mental health due to the cumulative effects of being exposed to experiences of stigmatisation, discrimination and fear of rejection from the wider population as well as others from each minority group [73–75]. Finally, the mapping exercise also revealed there to be a limited number of interventions targeted at carers. It is well known that caring can have an adverse impact on mental health which carers attribute to a lack of support [76]. This may indicate a gap in provision for this high-risk group or that services for carers do not explicitly state that they aim to improve the mental health and wellbeing of the carer and therefore were not captured by this study.

### COVID-19: implications for community PMH provision

The pandemic has likely impacted many determinants of PMH especially social isolation and loneliness, financial stress, job insecurity and unemployment, caring responsibilities, bereavement and gender-based violence [28–31]. The mapping exercise identified many interventions in place prior to March 2020 indicating the presence of expertise and infrastructure to respond to these challenges. However, the delivery of many public mental health support interventions was limited by lockdown measures [28], and likely affected the interventions mapped in this study. Digital technology enabled some interventions to continue to provide their services virtually during the pandemic [28], however, due to digital exclusion and inequality, access to such provision is unequal among residents [77, 78]. As this mapping exercise was undertaken prior to March 2020, we did not collect information regarding whether identified interventions have adapted or suspended their service provision.

The pandemic has not affected all communities equally, with high-risk sub-groups including: health and care workers; non-medical frontline workers (such as shop workers); members of Black, Asian and minority ethnic groups; people of lower or precarious incomes; people who have experienced COVID-19 or COVID-19-related bereavement; as well as older adults and people who have severe health conditions and had to “shield” for prolonged periods of time [28, 29, 31].

Given that almost half of the interventions identified in this mapping exercise were universal interventions, strategies need to be developed and implemented at national and local level to ensure the provision of timely and effective support to reduce or mitigate the risks of poor mental health among these higher risk groups [29–31]. Therefore, a shift toward targeting may urgently be needed for some part of the foreseeable future.

### Limitations

The primary limitation of the study is that it is difficult to assess how comprehensive the mapping was for each study site. It cannot be guaranteed that our community contacts were able to provide the most accurate and up-to-date information about interventions and services in their area and some local authority and third sector websites were out of date and incomplete; this is particularly pertinent given the fast-changing provision landscape resulting from the COVID-19 pandemic. Small community-based interventions (particularly those with a limited online presence – such as those organised by minority ethnic groups) may have been missed. Though we used multiple sources to identify and obtain information to minimize such limitations, detailed more community engaged exploration of community-funded organisations in particular is warranted in the future.

Only interventions and services that explicitly aimed to improve mental health or wellbeing were included, and we excluded wholly private-for-profit interventions. Work-based interventions and community focused initiatives by business are hence not captured. Many services positively impacting public mental health may not list mental health or wellbeing improvement as an explicit aim (e.g. a cycling group, a yoga course, a book club, choirs etc). This observation leads us to note that opportunities for collaborative and more effective working between mental health stakeholders and these services might currently be missed. We also excluded interventions aimed at children and adolescents, although we did include parenting interventions.

Inevitably, there was some subjectivity regarding how to define a “unit” of intervention, for example in the case of one small community group providing many different services. Equally, many small-scale social activity interventions might reach fewer people in total than one financial stress intervention reaching hundreds of people. As such, frequency data should be interpreted with some caution, and we suggest that patterns of relative provision are more insightful.

Lastly, the study was unable to consistently collect information on funding, programme numbers, and how long interventions had been running. This was potentially due to complexities surrounding the project-based nature of interventions where different funding streams begin and end at different stages within the life of the project. The study was also unable to collect information on the percentage of interventions that had been evaluated. This is because there were complexities within some organisations that delivered interventions. Either only part of a larger intervention had been evaluated or evaluations had focused on whether individuals had benefited from their overall involvement with an organisation, but each individual may have undertaken different individual interventions or a different combination of individual interventions within that organisation. It also should be considered that the existence of an intervention does not guarantee it is well designed or delivered, or effective at mitigating PMH risks. Indeed, the paucity of evaluation data for identified interventions means that this mapping study cannot determine their impact on PMH, only patterns of provision and localised prevalence of particular types. In future, policy-makers could address this heterogeneity of information about interventions and therefore enhance the availability of evaluation data, by making embedding standardised monitoring and evaluation in funding conditions, enabling more consistent or standardised funding streams, and/or providing better support for evaluations in the form of funding and/or expertise.

## Conclusions

This mapping exercise revealed evidence of some responsiveness to national and local prioritising. Service provision was geared towards addressing social and individual determinants of PMH, with broader interventions focusing on structural and environmental determinants uncommon. This suggests more integration is needed to engage broader stakeholders in PMH strategy and delivery. Specifically, we recommend that PMH impact assessments should be embedded in these broader interventions to increase the body of evidence on the effectiveness of community interventions to improve adult PMH. The mapping exercise identified many interventions that attempted to address mental health inequalities by targeting specific groups in the community such as older adults, people from minority ethnic backgrounds, women, men, carers and LGBTQ+ populations. However, some at-risk populations may have been missed or underserved, for example men from minority ethnic backgrounds and LGBTQ+ people from minority ethnic backgrounds.

In-depth evaluations of the effectiveness of community-based interventions are still required, especially focusing on the relevance of the context of service delivery and how stakeholders work together operationally. Policy makers and researchers, in future, could also focus on how providers of community-based projects can be better supported to evaluate the effectiveness of both their interventions and their approaches to delivering within dynamic community-based systems, and to share data more effectively across projects and sectors.

## Supplementary Information

**Additional file 1.** Data extracted about each intervention, if available (list adapted from TiDieR Checklist). This additional file provides information about the type of data that we aimed to extract for each of the interventions identified in this mapping exercise.

## Data Availability

All data generated or analysed during this study are included in this published article.

## Abbreviations

PMH: Public Mental Health
ONS: Office of National Statistics
IoD: Indices of Deprivation
IMD: Index of Multiple Deprivation
LSOA: Lower-layer super output area
TIDieR: Template for Intervention Description and Replication
NHS: National Health Service
NIHR: National Institute of Health Research
SPHR: School for Public Health Research
LGBTQ+: Lesbian, gay, bisexual and transgender
UK: United Kingdom
