# Supplementary material for: Community interventions for improving adult mental health: mapping local policy and practice in England
Source: BMC Public Health. 2021 Sep 16;21:1691. doi: 10.1186/s12889-021-11741-5 (PMC8444510; doi:10.1186/s12889-021-11741-5)
Supplement: Supplementary file 1 — Additional file 1. Data extracted about each intervention, if available (list adapted from TiDieR Checklist). This additional file provides information about the type of data that we aimed to extract for each of the interventions identified in this mapping exercise. [file 12889_2021_11741_MOESM1_ESM.docx]

**Additional file – Data extracted about each intervention, if available (list adapted from TiDieR Checklist).**

| - Type of intervention |
| --- |
| - Aims and objectives (i.e. which problem or risk factor does it seek to address?) |
| - Target population (who is eligible to participate) |
| - Intended primary and secondary outcomes |
| - Content of intervention (which activities, procedures and processes take place, as well as the schedule, duration and intensity of delivery) |
| - Geographical area of delivery |
| - Setting of delivery (what facilities, infrastructure and support are required to deliver the intervention) |
| - Method of delivery (online, face-to-face, in groups) |
| - Who delivers the interventions (expertise, background, training) |
| - Who is the provider of the intervention? |
| - How is it funded? |
| - What is the funding period? |
| - Has the intervention been evaluated? |
